# A New Model for Optimal Mechanical and Thermal Performance of Cement-Based Partition Wall

**DOI:** 10.3390/ma11040615

**Published:** 2018-04-17

**Authors:** Shiping Huang, Mengyu Hu, Yonghui Huang, Nannan Cui, Weifeng Wang

**Affiliations:** 1 School of Civil Engineering and Transportation, South China University of Technology, Guangzhou 510640, China; ctasihuang@scut.edu.cn (S.H.); 201620105537@mail.scut.edu.cn (M.H.); wfwang@scut.edu.cn (W.W.); 2 State Key Laboratory of Coal Resources and Safe Mining, China University of Mining and Technology, Xuzhou 221116, China; 3 Guangzhou University-Tamkang University Joint Research Center for Engineering Structure Disaster Prevention and Control, Guangzhou University, Guangzhou 510006, China; 4 School of Transportation Engineering, Shandong Jianzhu University, Jinan 250101, China; cuinannan18@sdjzu.edu.cn

**Keywords:** porous materials, mechanical properties, cement-based materials, thermal analysis, partition wall, finite element method

## Abstract

The prefabricated cement-based partition wall has been widely used in assembled buildings because of its high manufacturing efficiency, high-quality surface, and simple and convenient construction process. In this paper, a general porous partition wall that is made from cement-based materials was proposed to meet the optimal mechanical and thermal performance during transportation, construction and its service life. The porosity of the proposed partition wall is formed by elliptic-cylinder-type cavities. The finite element method was used to investigate the mechanical and thermal behaviour, which shows that the proposed model has distinct advantages over the current partition wall that is used in the building industry. It is found that, by controlling the eccentricity of the elliptic-cylinder cavities, the proposed wall stiffness can be adjusted to respond to the imposed loads and to improve the thermal performance, which can be used for the optimum design. Finally, design guidance is provided to obtain the optimal mechanical and thermal performance. The proposed model could be used as a promising candidate for partition wall in the building industry.

## 1. Introduction

The optimum design of the prefabricated components is key to energy savings, environmental protection, and the maximum life of building products [1,2,3]. In recent years, the prefabricated cement-based partition wall has been widely used in assembled buildings because of its high manufacturing efficiency, high-quality surface, and simple and convenient construction process. In an assembled building, the partition wall is up to 50% of the total weight of the entire structure, even though it does not participate as a structural member. To reduce the weight, porous partition walls with cavities have been widely used in recent years. Due to the existence of the cavities, the partition wall, similar to other porous structures [4,5,6,7], is often subjected to complex stress conditions under external loads. Furthermore, the thermal performance of the partition wall is also an important factor in the design. Therefore, the focus of this work is to provide a better model to obtain the optimal mechanical and thermal performance.

Most previous work has focussed on the single function performance of the partition wall, such as material properties, seismic behaviour [8,9], sound insulation, and thermal properties [10,11,12,13], while the mechanical and thermal properties have not been fully investigated for all the possible loadings, which may cause cracking and reduce the function during a wall’s service life. Understanding of the mechanical and thermal properties for all the possible loadings is essential to get the optimal mechanical and thermal performance (performance based multi-objective optimization [14,15]) for the design. There are many new materials used for partition wall, which have a direct effect on the wall’s mechanical and thermal performance. However, the wall’s geometry effect on the mechanical and thermal performance is more complicated and needs further study. The purpose of this work is to propose a new general geometry model for the partition wall, which can obtain the optimal mechanical and thermal performance.

In the following section, we will first present a general porous partition wall with elliptic-cylinder cavities and then study the effect of geometry on the mechanical-thermal performance using the finite element method. In particular, a comparison is made with a current design that is used in the building industry. Finally, simple guidance is provided to obtain the mechanical and thermal performance, which can be used for the optimum design.

## 2. Materials and Methods 

### 2.1. Geometry of the Partition Wall

**Figure 1:**
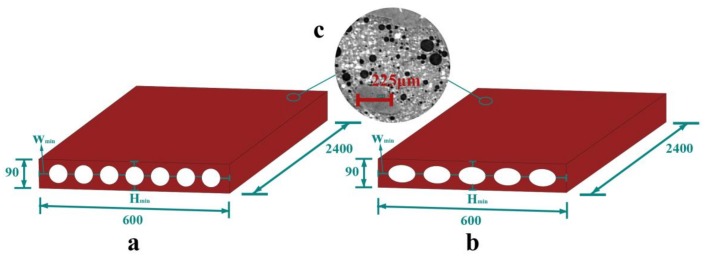
Dimensions of the partition wall (in mm): (**a**) current model used in the building industry; (**b**) proposed model; and, (**c**) microstructure of the cement-based materials.

Recently, in the building industry, a prefabricated porous partition wall that was recommended by the specification [16,17] is widely used, in which the porosity is formed by seven circular cylinder cavities, as shown in Figure 1. The partition wall is assembled by the tenon and mortise joints that are left on the edges, and then the tenon and mortise joints are coated with glue and cement. To reduce the stress concentration zones at the cavity boundaries and to improve the thermal performance, we propose a more general porous structural model, i.e., using five elliptical cylinders to form the porous structure, as seen in Figure 1b. It is noted that the circular cavity partition wall that is used in the industry now is a special case of the proposed model. There are two obvious advantages of this geometry: (1) the reduced number of cavities can make the fabrication easier; and, (2) the spacing can be controlled by the eccentricity, which can further control the mechanical-thermal performance. The eccentricity is defined by $e=\sqrt{1-b^{2}/a^{2}}$, where *a* is the major axis and *b* is the minor axis for the ellipse.

The partition wall consists of cement-based materials [18,19] and a small amount of reinforcement (less than 1%). It is noted that the proposed model is not limited to cement-based materials and it can use other materials [20,21,22,23]. A popular fabrication process that is used for the partition wall is compression moulding. Even though the cement-based materials exhibit multiscale porosity behaviour [24,25,26,27,28,29,30,31] (as seen in Figure 1), by using the homogenization method [32,33,34], it can be modelled as continuum materials by choosing the proper material parameters. The strength of the partition wall is usually between 10–15 MPa, which is similar to Chinese C10–C15 concrete. Here, the numerical simulation is based on Chinese C15 concrete, whose density is 2400 kg/m^3^, the elastic modulus is 2.2 × 10^4^ N/mm^2^ and Poisson’s ratio is 0.16. To study the eccentricity and the porosity effect on the mechanical-thermal properties, 13 specimens for the numerical simulations are designed, and the dimensions are shown in Table 1.

### 2.2. Boundary Conditions for the Numerical Analysis

**Figure 2:**
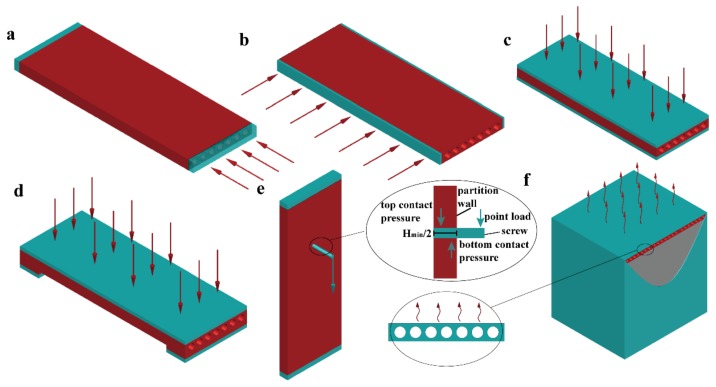
Boundary conditions of the partition wall (load 2a–load 2f): (**a**–**c**) uniformed loading in different directions; (**d**) bending under the uniformed loads; (**e**) hanging test boundary condition; and, (**f**) heat transfer boundary condition.

**Figure 3:**
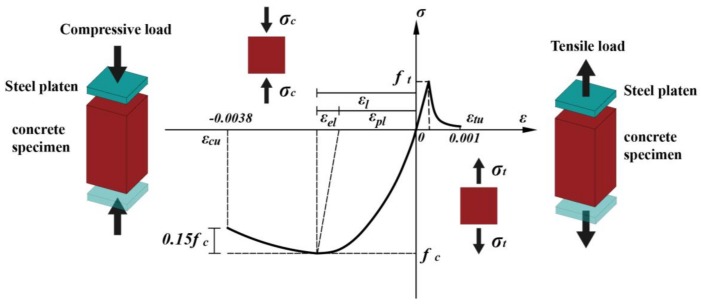
Hognestad model [40] and Mazars model [41] of the cement-based materials that were used in the simulation.

**Figure 4:**
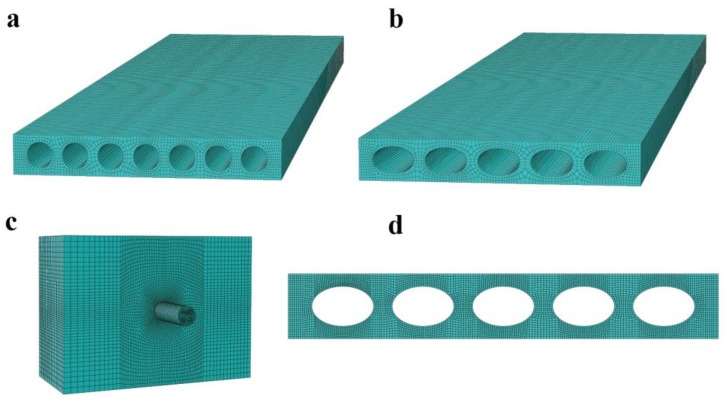
Finite element (FE) models for the partition walls: (**a**) Circular cavity panel in a three-dimensional (3D) view of the FE model; (**b**) Elliptical cavity panel in a 3D view of the FE model; (**c**) FE model of hanging test; and, (**d**) two-dimensional (2D) FE model of thermal analysis.

The stress distribution is very complex for a porous structure under external loading. An elastic-plastic response provides the stress distribution (the stress concentration or maximum stress are key factors) and the ultimate strength. To this end, we first use the finite element method [35,36,37,38] to study the elastic-plastic response under specific loads that may occur during transportation, construction and its service life. The partition panel will be subjected to the following load conditions during the construction and when the board is in service: (1) the uniform displacement transferred from the top beam after installation, as in Figure 2a; (2) the squeezing force between the adjacent partition walls on the side, as in Figure 2b; (3) the uniform displacement applied on the plane during transportation or packing, as in Figure 2c; (4) bending caused by a uniform pressure during transportation and service, as in Figure 2d; and, (5) contact forces caused by hanging during service in the most disadvantageous position (at location *H_min_*), as in Figure 2e. The boundary conditions in Figure 2a–f are as follows: Figure 2a–c demonstrate that one side is applied uniform displacement while the other side is fixed; Figure 2d demonstrates that the top side is applied with uniform pressure in a simply supported beam boundary condition; Figure 2e demonstrates that one side is fixed, while the screw is under a point load.

Furthermore, to simulate the thermal performance of different partition panels, a space of three cubic metres is designed, in which one side is assembled by a partition wall, and the other sides use thermal insulation materials, as shown in Figure 2f. In Figure 2f, the inner temperature is 25 degrees Celsius, while the exterior temperature is 0 degree Celsius, and the exterior wind speed is set to be 5 m/s. The heat flow at the minimum width location *W_min_* is used to evaluate the thermal performance. The numerical details are discussed in the following section.

### 2.3. Numerical Methods

The mechanical and thermal behaviour was investigated by the finite element method. For the completeness, the detailed mathematical models were described in the following sections, which were then carried out in ABAQUS [39].

#### 2.3.1. Materials Response in the Uniaxial Tests

The Hognestad model [40] (as shown in Figure 3) is used for the uniaxial compressive response of the material, which is given as:

(1) $$\begin{array}{l} for(0\leq \varepsilon \leq \varepsilon_{0}):\sigma =f_{c}[2\frac{\varepsilon }{\varepsilon_{0}}-{\left(\frac{\varepsilon }{\varepsilon_{0}}\right)}^{2}]\\ for(\varepsilon_{0}\leq \varepsilon \leq \varepsilon_{{}_{cu}}):\sigma =f_{c}\left[1-0.15\frac{\varepsilon -\varepsilon_{0}}{\varepsilon_{cu}-\varepsilon_{0}}\right] \end{array}$$

where *σ* and *ε* are the stress and strain during uniaxial testing and *f_c_* is taken as 7.2 MPa in our simulation. The Mazars model [41] (as shown in Figure 3) is used for uniaxial tensile response of the material, which is given as:

(2) $$\begin{array}{l} for(0\leq \varepsilon <\varepsilon_{f}):\sigma =E_{0}\varepsilon \\ for(\varepsilon_{f}\leq \varepsilon \leq \varepsilon_{tu}):\sigma =E_{0}[\varepsilon_{f}(1-A_{T})+\frac{A_{T}\varepsilon }{\exp[B_{T}(\varepsilon -\varepsilon_{f})]}] \end{array}$$

where 0.7 ≤ *A_T_* ≤ 1, 10^4^ ≤ *B_T_* ≤ 10^5^, and in this simulation, *A_T_* = 0.9, *B_T_* = 2 × 10^4^, *ε_f_* = 5.8 × 10^−5^.

#### 2.3.2. Elastic Finite Element Method

The partition wall is considered as continuum materials, where the reinforcement is neglected. Without considering the body force, the stress field satisfies the following equation:

(3) $$\sigma_{ij,j}=0$$

The boundary condition is written as:

(4) $$t_{i}=\sigma_{ji}n_{j}$$

where $\sigma_{ij}$ is the stress tensor; *i*, *j* are the subscripts of the stress tensor, indicating the *x*, *y*, *z* directions; $t_{i}$ is the external surface load; and, *n_j_* is the normal vector. The weak form of the governing Equation (3) can be expressed as:

(5) $$\int_{\Omega }\sigma_{ij,j}\delta u_{i}^{}dV=0$$

Using the divergence theory and Equation (3), Equation (5) can be expressed as:

(6) $$\int_{V}\sigma_{ij}\delta u_{i,j}^{}dV=\int_{S}t_{i}\delta u_{i,j}^{}dS$$

Note that Equation (6) can also be achieved by the principle of virtual work, i.e., the virtual work of the external forces is equal to the virtual change in the internal energy. The finite element method discretizes the entire domain into a number of elements, and each element contains several nodes. The displacement *u_i_* is expressed via the element node displacement *u_i_^n^* as:

(7) $$u_{i}=\varphi_{n}u_{i}^{n}$$

(8) $$\int_{V_{e}}\sigma_{ij}\frac{\partial \varphi_{n}}{\partial x_{j}}u_{i}^{n}dV=\int_{S_{e}}t_{i}\varphi_{n}u_{i}^{n}dS$$

Since the virtual displacement is arbitrary, we finally obtain the equilibrium equation for each element:

(9) $$\begin{array}{l} \left[K_{e}\right]\left[U_{e}\right]=\int_{V_{e}}\sigma_{ij}\frac{\partial \varphi_{n}}{\partial x_{j}}u_{i}dV\\ \left[F_{e}\right]=\int_{S_{e}}t_{i}\varphi_{n}dS \end{array}\}$$

After assembling the element for the entire domain, the Equation (9) will have the final form [*F*] = [*K*][*U*], where [*F*] is the node’s force, [*K*] is the stiffness matrix, and [*U*] is the node’s displacement. In the finite element analysis (FEA), which is a hexagonal eight-node element, is used for mechanical analysis and a 4-node linear heat transfer quadrilateral element is used for two-dimensional (2D) thermal analysis, as seen in Figure 4.

#### 2.3.3. The Incremental Elastic-Plastic Constitutive Model

During the plastic loading stage, the loads are discretized into finite increments, and for each increment, the constitutive behaviour is considered to be linear. Moreover, the total strain increment is expressed as the sum of the elastic and plastic strain increments, i.e.,

(10) $$d\varepsilon_{ij}=d\varepsilon_{ij}^{e}+d\varepsilon_{ij}^{p}$$

If we consider the cement-based material as an isotropic material, the incremental stress-strain relationship can be expressed as:

(11) $$d\sigma_{ij}=C_{ijkl}(d\varepsilon_{kl}-d\varepsilon_{ij}^{p})$$

where *C_ijkl_* is the stiffness tensor, which is given as:

(12) $$C_{ijkl}=\frac{E_{0}}{1+v}(\delta_{ik}\delta_{jl}+\frac{v}{1-2v}\delta_{ik}\delta_{jl})$$

where *E*_0_ and *v* are the Young’s modulus and Poisson’s ratio, respectively. Following the plasticity theory, the relationship of the stress increment and the total strain increment for the Von Mises material can be written as:

(13) $$d\sigma_{ij}=\left(C_{ijkl}-\frac{\frac{\partial f}{\partial \sigma_{rs}}C_{ijrs}C_{mnkl}\frac{\partial f}{\partial \sigma_{mm}}}{\frac{\partial f}{\partial \sigma_{ab}}C_{abcd}\frac{\partial f}{\partial \sigma_{cd}}}\right)d\varepsilon_{kl}=C_{ijkl}^{ep}d\varepsilon_{kl}$$

Without considering the body force, the final governing equation in terms of incremental elastic-plastic constitutive equation is given as:

(14) $$\int_{V}C_{ijkl}^{ep}d\varepsilon_{kl}\delta (d\varepsilon_{ij})dV-\int_{S_{\sigma }}d\bar{T_{i}}\delta (u_{i})dS+\int_{V}\sigma_{ij}\delta (d\varepsilon_{ij})dV-\int_{S_{\sigma }}\bar{T_{i}}\delta (du_{i})dS=0$$

where *δ* indicates the variational operator and *S_σ_* and *T_i_* are the area and boundary stress vectors, respectively. Again, Equation (13) is discretized into a number of elements and follows the procedure to establish the finite element model. Equation (13) is readily solved by the commercial finite element software ABAQUS.

#### 2.3.4. Computational Contact Theory

**Figure 5:**
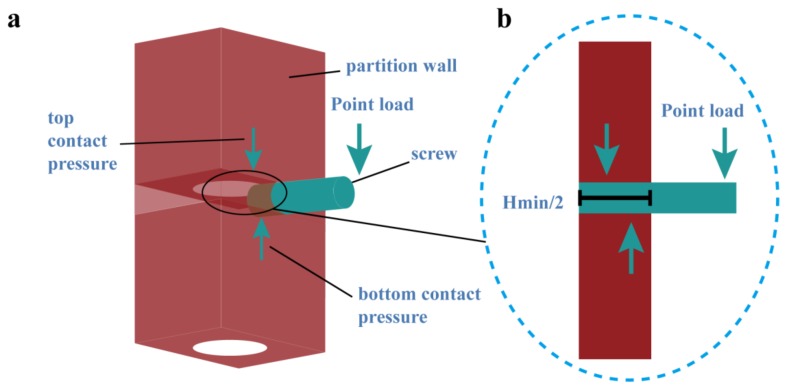
Finite element model for the contact of a screw and the wall during hanging: (**a**) screw located at the most disadvantageous position (at location *H_min_*); and, (**b**) equilibrium of the contact force and the external force.

Hanging objects on a wall is one of the basic functions for a partition wall. Here, we are using the contact theory to simulate the contact force between the wall and the screw when the screw is under a bending moment, as seen in Figure 5. For simplicity, the screw is modelled as a cylinder. When the screw is under a point load as a cantilever, the reaction contact force occurs to maintain the equilibrium. In this case, the tension force between the screw and the concrete can be negligible. Without considering the adhesion and the friction, the contact boundary condition is as follows:

(15) $$g_{n}\sigma_{n}=0,g_{n}\geq 0,\sigma \leq 0,\sigma_{n}=\sigma \cdot n$$

where *g_n_* is the gap between the contact solids and *σ_n_* is the contact pressure. Then, the weak form of the governing Equation (3) is changed to the following form:

(16) $$\int_{V^{1,2}}\sigma_{ij,j}\delta u_{i}^{}-\int_{S^{1,2}}t_{i}\delta u_{i}^{}dT-\int_{S^{c}}\sigma_{n}\delta g_{n}dT=0$$

Since the contact area is changing during the analysis, the problem is nonlinear, and the linearization process is similar to that in Section 2.3.3, where the loading is divided into many increments. The finite element algorithm can be applied to solve the weak form by using the Lagrange amplifier method, which is performed by ABAQUS.

#### 2.3.5. Thermal Analysis

According to the theory of Fourier heat conduction, the three-dimensional heat conduction equation is given as:

(17) $$\lambda \left(\frac{\partial^{2}T}{\partial x^{2}}+\frac{\partial^{2}T}{\partial y^{2}}+\frac{\partial^{2}T}{\partial z^{2}}\right)=c\gamma \frac{\partial T}{\partial t}$$

where *λ* is the thermal conductivity (1.28 W/(m·K)); *γ* is the density of the concrete (2400 kg/m^3^); and *c* is the specific heat capacity (920 J/(kg·K)). If the temperature is uniform along the height direction, then the three-dimensional (3D) problem can be reduced to a 2D problem. The boundary condition for the heat exchange is as follows:

(18) $$\lambda \left(\frac{\partial T}{\partial n}\right)=\beta (T_{1}-T_{2})$$

where *β* is the convection heat transfer coefficient and *n* is the normal vector. The convection heat transfer coefficient is affected by many factors, such as the angle and speed of the wind [42]. Here, we use the result based on a wind tunnel experimental study [43], which is given by the following equation:

(19) β=3.06v+4.11

where *v* is the wind speed close to the concrete partition wall surface, which is taken as 5 m/s (a gentle breeze that mostly occurs in our daily life). The weak form of the governing Equation (17) can be expressed as:

(20) $$\int_{V}\lambda (\frac{\partial^{2}T}{\partial x^{2}}+\frac{\partial^{2}T}{\partial y^{2}}+\frac{\partial^{2}T}{\partial z^{2}})\delta T-c\gamma \frac{\partial T}{\partial t}\delta TdV=0$$

In the finite element model, similar to the discussion in Section 2.3.2, the temperature field *T* is represented by the test function via the node’s temperature. The finite element algorithm ABAQUS is used to solve the above equation numerically.

## 3. Results and Discussions

### 3.1. Mechanical Properties

#### 3.1.1. Effect of the eccentricity

**Figure 6:**
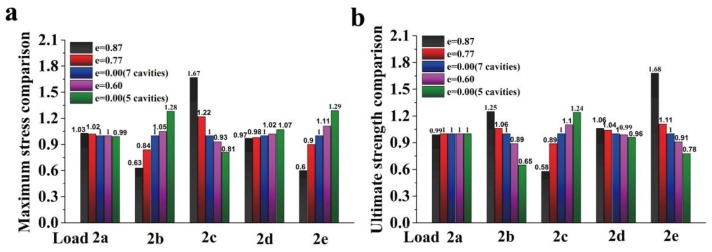
Effect of the eccentricity on the mechanical properties: (**a**) maximum stress comparison under elastic loading, and (**b**) ultimate strength comparison under failure simulation.

**Figure 7:**
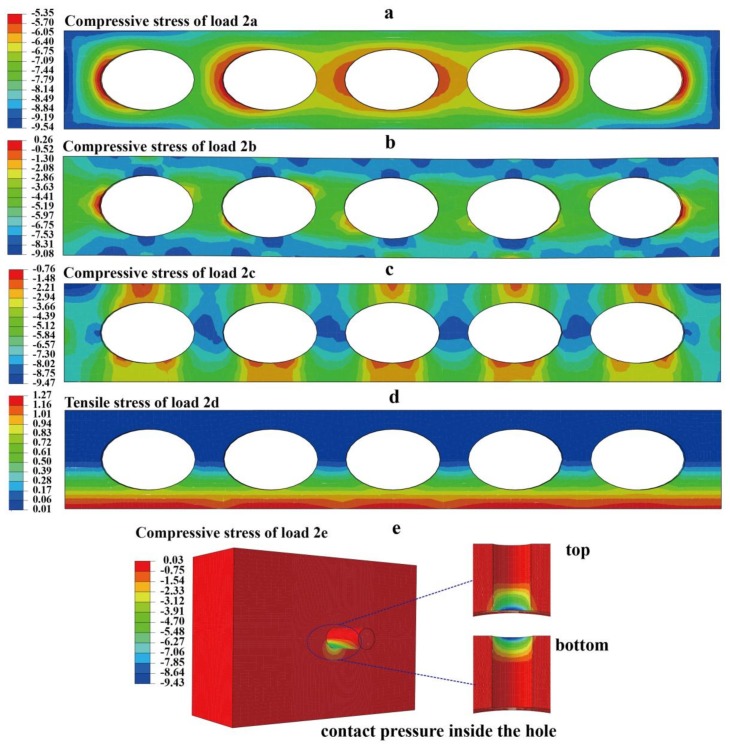
Stress distribution at the failure simulation (in MPa): (**a**–**e**) stress contours under failure simulation for the five loading conditions, according to Figure 2a–e.

To study the eccentricity effect on the mechanical properties, four different elliptical cavity panels, i.e., e = 0, 0.6, 0.77, and 0.87, are used as in Table 2. For comparison, the current circular cavity (seven cavities) partition wall is used. Currently, the porosity that is used for the partition wall is approximately 35%, which is also utilized in this analysis. The mechanical response of the elastic-plastic loading was carried out to study the partition wall. The Hognestad model (uniaxial compressive response) [40] and Mazars model (uniaxial tensile response) [41] are used for the elastic-plastic constitutive law. It is noted that the ultimate structural strength (referred to as strength, which is equal to the ultimate force divided by nominal area) is the peak value extracted from the displacement-load curve. In Figure 6a,b, the maximum stress comparison ratios (ultimate strength ratio) that were obtained by normalizing the maximum stress (ultimate strength) with that from the circular cylinder model are shown for different loading conditions. Maximum stress comparison ratio is referred to as normalize ratio in Table 2 and Figure 6. For the loading case in Figure 2a, the maximum stress ratios for the different eccentricities are almost the same. This is because the cavity’s direction is parallel to the loading direction. In this case, the strength is dominated by the actual area (they are the same because the porosities are the same) since the stress distribution tends to uniform for regions that are far from the boundary due to the Saint Venant’s Principle. For the loading case in Figure 2b,d,e, the larger the eccentricity, the smaller maximum stress ratio. However, in the loading case of Figure 2c, the larger eccentricity leads to a smaller maximum stress ratio. The opposite trends are observed for the ultimate strength ratios. Even though the trade-off was made in the Figure 2c loading, this loading condition seldom appears. These results suggest that for the same number of cavities with acceptable strength during the packing (Figure 2c), a larger eccentricity leads to better mechanical performance and design. The contour of the failure stress is shown in Figure 7a–e, and the failure stress tends to more uniform than that from elastic loading due to the plastic flow. To verify the numerical results, a typical experiment was carried out for the specimen CP (circular cavity partition, made from materials based on Chinese C15 concrete) under the Figure 2a loading case. In the experiment, 30 specimens have been tested and the average structural strength is 5.46 MPa with standard deviation of 0.75 MPa. When comparing with the numerical result 4.68 MPa for CP, the average strength is 16.7% larger than that of the numerical result. This is acceptable for the concrete structures, whose experimental results are usually more discrete than the other materials. This suggests that the finite element model in this work can get quite reasonable results in a small amount of time.

#### 3.1.2. Effect of porosity

**Figure 8:**
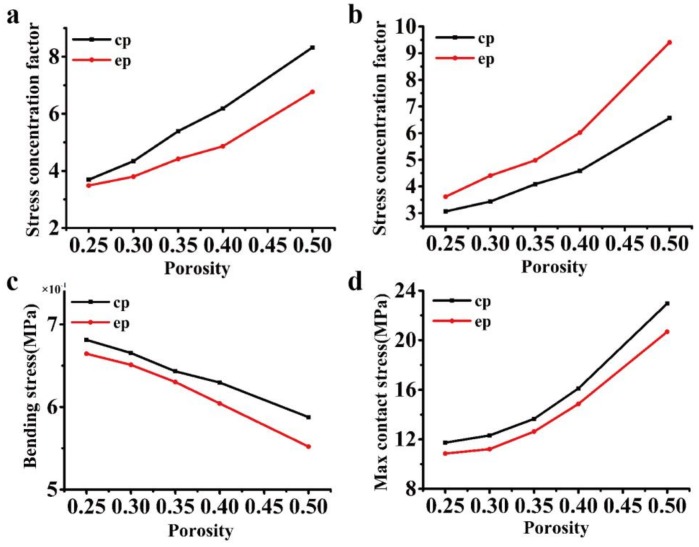
Comparison of the stress concentration factor under different porosities: (**a**–**d**) stress concentration factor according to the loading cases in Figure 2b–e.

**Figure 9:**
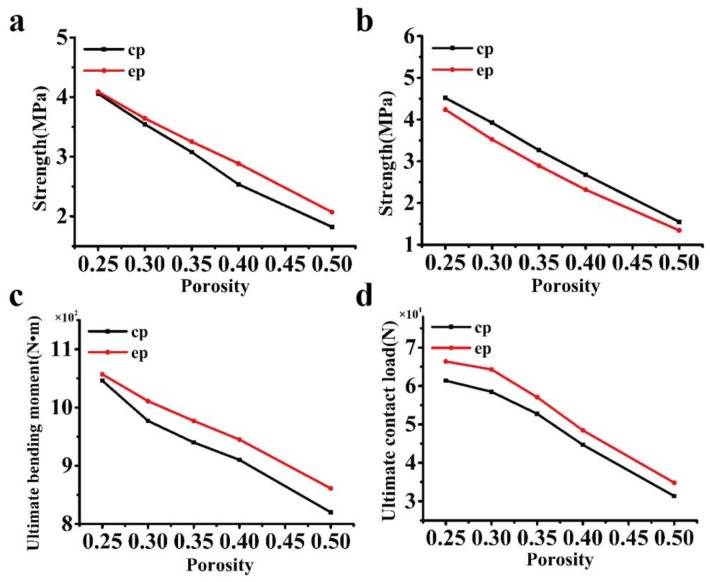
Comparison of the ultimate strength under different porosities: (**a**–**d**) ultimate strength according to the loading cases in Figure 2b–e.

To investigate the effect of porosity on the mechanical properties (in Figure 8 and Figure 9), the eccentricity is set at 0.77, while the porosity changes from 25–50%, as seen in Table 3 and Table 4. Again, the elliptical cavity model is compared with the circular cavity model that is used in industry. The stress concentration factor is compared for the loading cases in Figure 2a–c. It is found that as the porosity increases, the stress concentration factor increases, while the ultimate strength decreases. For the loading case in Figure 2a, the proposed model has nearly the same stress concentration factor as the cylinder cavity model that is used in industry (the reason is discussed in Section 3.1.1), which is not shown in Figure 8. For the loading case in Figure 2b,d,e, the elliptical cavity model exhibits a better performance than that of the circular cavity model, i.e., it has a smaller stress concentration or bending stress and a higher strength, whereas in the loading case in Figure 2c, the circular cavity model that is used in industry has better performance, as seen in Figure 8 and Figure 9.

### 3.2. Thermal Performance

**Figure 10:**
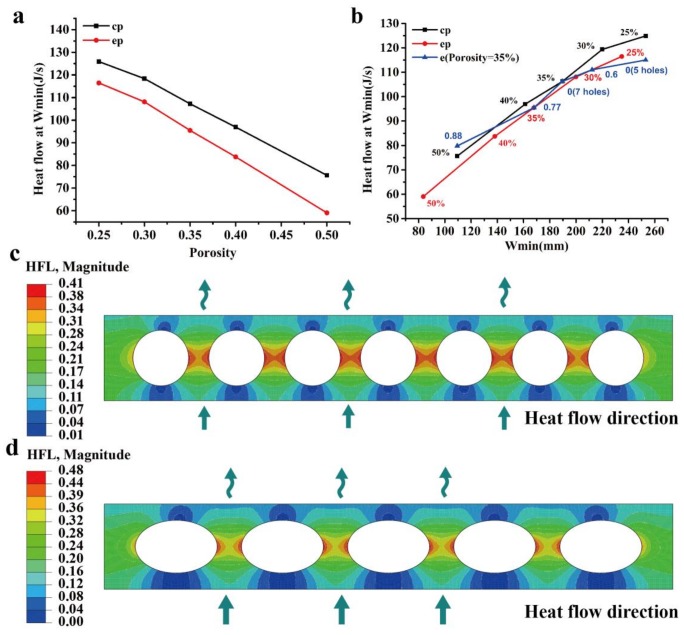
Porosity and minimum width effect on the heat flux and heat flow (in J/s): (**a**) porosity effect on the heat flow; (**b**) minimum width effect on the heat flow; and, (**c**,**d**) contour of the heat flux distribution.

The heat flow and heat flux (HFL) are important parameters to evaluate the thermal performance of the structure. It is noted that the heat flow is the sum of the heat flux passing through a section. As seen in Table 5, Figure 10a,b, it is found that the heat flow at the location *W_min_* increases with the minimum width and decreases with the porosity. For a given boundary condition (heat transfer coefficient and temperature difference), it is observed that the heat flow is almost linearly related to the minimum width and porosity. However, this may be affected by the boundary condition, since the heat flux is not uniformly distributed along with the width (in Figure 10c,d) and it can be affected by different boundary conditions. For the same porosity, the minimum width in the proposed model is smaller (the heat flow lower) than that of the cylinder cavity model, which indicates that the thermal performance is better. In particular, at a porosity of 35%, the heat flux in the proposed model (e = 0.77) is 10% lower than that of the cylinder cavity model, which indicates that the proposed model demonstrates an approximately 10% energy savings. As the eccentricity increases, the proposed model has better thermal performance than that of the circular cavity partition wall at the same porosities, as shown in Table 5. The numerical results show a good agreement with the numerical and the experimental results in the literature [44,45,46]. Generally, for the optimum design, a smaller porosity leads to smaller weight but weaker strength of the partition wall. Once the porosity and the packing strength (loading of Figure 2c) have been determined, a larger eccentricity leads to a better mechanical and thermal performance.

## 4. Conclusions

A general porous model is proposed for a prefabricated partition wall. The porosity is formed by elliptical cylinders and the circular cavity partition wall becomes a special case of the proposed model. The wall’s geometry effect on the mechanical and thermal performance has been investigated by the finite element method and typical experiments. It is found that, by controlling the eccentricity, the model can adjust the stiffness for different loading directions. When compared to the partition wall with regular circular cavities, the proposed model increases the strength in most loading conditions, such as a squeezing force from the top and the sides and the bending and contact forces that are caused by hanging. Due to the reduction in the minimum width, the proposed model exhibits a better thermal performance. With an acceptable strength during the packing, a larger eccentricity leads to better mechanical and thermal performance for the same porosity and cavity number. This can be used as guidance for the optimal design. The proposed model also provides a smaller number of the hollow zones, which decrease the stress concentration zones and facilitates the fabrication. The proposed model could be used as a promising candidate for partition wall in the building industry.

## Figures and Tables

**Table 1:** Dimensions of the specimens.

Specimen	CP	CP1	CP2	CP3	CP4	EP	EP1	EP2	EP3	EP4	CP5	EP5	EP6
major axis (mm)	29	25	27	31	35	43	37	40	46	52	35	49	39
minor axis (mm)	29	25	27	31	35	28	24	26	30	33	35	25	31
eccentricity	0	0	0	0	0	0.77	0.77	0.77	0.77	0.77	0	0.87	0.60
porosity	35%	25%	30%	40%	50%	35%	25%	30%	40%	50%	35%	35%	35%
number of cavities	7	7	7	7	7	5	5	5	5	5	5	5	5
minimum width (mm)	190	253	220	161	109	168	235	200	138	84	253	109	212

**Table 2:** Effect of the eccentricity on the mechanical properties.

Specimen	CP	EP	CP5	EP5	EP6
eccentricity	0	0.77	0	0.87	0.6
porosity	35%	35%	35%	35%	35%
stress concentration factor (load 2a)	2.01	2.04	1.99	2.07	2.02
Normalized Ratio (load 2a)	1.00	1.02	0.99	2.07	2.05
ultimate strength (load 2a) (MPa)	4.68	4.67	4.68	4.65	4.67
Normalized Ratio (load 2a)	1.00	1.00	1.00	0.99	1.00
stress concentration factor (load 2b)	5.39	4.42	6.91	3.39	5.65
Normalized Ratio (load 2b)	1.00	0.84	1.28	0.63	1.08
ultimate strength (load 2b) (MPa)	3.08	3.25	1.99	3.84	2.74
Normalized Ratio (load 2b)	1.00	1.06	0.65	1.25	0.89
stress concentration factor Ratio (load 2c)	4.08	4.98	3.31	6.83	3.79
Normalized Ratio (load 2c)	1.00	1.22	0.81	1.67	0.93
ultimate strength (load 2c) (MPa)	3.27	2.90	4.05	1.89	3.59
Normalized Ratio (load 2c)	1.00	0.89	1.24	0.58	1.10
Stress under bending (load 2d) (MPa)	0.64	0.63	0.69	0.62	0.65
Normalized Ratio (load 2d)	1.00	0.98	1.07	0.97	1.02
ultimate bending load (load 2d) (N·m)	941.25	977.92	905.67	1001.85	936.59
Normalized Ratio (load 2d)	1.00	1.04	0.96	1.06	0.99
contact stress (MPa) (load 2e)	13.65	12.62	18.01	8.33	15.43
Normalized Ratio (load 2e)	1.00	0.90	1.29	0.60	1.11
the ultimate contact strength (load 2e) (N)	52.75	57.05	39.98	86.49	46.66
Normalized Ratio (load 2e)	1.00	1.11	0.78	1.68	0.91

**Table 3:** Comparison of the stress concentration factor under different porosities.

Specimen	CP	CP1	CP2	CP3	CP4	EP	EP1	EP2	EP3	EP4
eccentricity	0	0	0	0	0	0.77	0.77	0.77	0.77	0.77
porosity	35%	25%	30%	40%	50%	35%	25%	30%	40%	50%
stress concentration factor (load 2a)	2.01	1.78	1.88	2.2	2.64	2.04	1.78	1.9	2.22	2.62
stress concentration factor (load 2b)	5.39	3.69	4.34	6.19	8.32	4.42	3.49	3.79	4.86	6.76
stress concentration factor (load 2c)	4.08	3.06	3.43	4.59	6.57	4.98	3.61	4.41	6.02	9.41
maximum stress under bending (kN·m)	0.64	0.68	0.67	0.63	0.59	0.63	0.66	0.65	0.60	0.55
contact stress (MPa)	13.65	11.73	12.31	16.11	22.96	12.62	10.85	11.20	14.86	20.68

**Table 4:** Comparison of the ultimate strength under different porosities.

Specimen	CP	CP1	CP2	CP3	CP4	EP	EP1	EP2	EP3	EP4
eccentricity	0	0	0	0	0	0.77	0.77	0.77	0.77	0.77
porosity	35%	25%	30%	40%	50%	35%	25%	30%	40%	50%
ultimate strength (load 2a) (MPa)	4.68	5.42	5.05	4.33	3.59	4.67	5.40	5.03	4.31	3.60
ultimate strength (load 2b) (MPa)	3.08	4.06	3.54	2.54	1.82	3.25	4.09	3.64	2.89	2.07
ultimate strength (load 2c) (MPa)	3.27	4.52	3.92	2.68	1.55	2.90	4.24	3.52	2.32	1.34
ultimate bending load (N·m)	941.25	1046.85	977.64	913.52	819.67	977.92	1057.66	1011.18	944.96	960.78
the ultimate contact strength (N)	52.75	61.38	58.49	44.69	31.36	57.05	66.36	64.29	48.45	34.82

**Table 5:** Comparison of the heat flow under different porosities.

Specimen	CP	CP1	CP2	CP3	CP4	EP	EP1	EP2	EP3	EP4	CP5	EP5	EP6
eccentricity	0	0	0	0	0	0.77	0.77	0.77	0.77	0.77	0	0.87	0.6
porosity	35%	25%	30%	40%	50%	35%	25%	30%	40%	50%	35%	35%	35%
Heat flow (J/s)	106.25	124.89	119.37	96.94	75.63	95.50	116.47	108.09	83.75	59.05	115.01	79.78	111.04

## Supplementary Materials

Supplementary File 1
