# Supplementary material for: A New Model for Optimal Mechanical and Thermal Performance of Cement-Based Partition Wall
Source: Materials (Basel). 2018 Apr 17;11(4):615. doi: 10.3390/ma11040615 (PMC5951499; doi:10.3390/ma11040615)
Supplement: Supplementary File 1 [file materials-11-00615-s001.pdf]

Towards optimal mechanical and thermal performance of cement-based partition wall

Supplementary Materials

More detailed Results

1.1 Effect of the eccentricity

Table S1: stress concentration factor under the loading based on Fig.2a-2c for different eccentricities.

porosity	35%				
eccentricity	0.87	0.77	0.6	0(5 hole)	0(7 hole)
semi-major axis(mm)	49.06	43.21	38.78	34.69	29.32
semi-minor axis(mm)	24.53	27.83	31.03	34.69	29.32
minimum width(mm)	109.45	167.91	212.18	253.13	189.57
minimum height(mm)	40.94	34.35	27.95	20.63	31.37
uniformed displacement applied to Fig.2a (mm)	1.00	1.00	1.00	1.00	1.00
stress concentration factor of Fig.2a	2.07	2.05	2.02	1.99	2.01
Normalized Ratio r_i	1.03	1.02	1.00	0.99	1.00
uniformed displacement applied to Fig.2b(mm)	1.00	1.00	1.00	1.00	1.00
stress concentration factor of Fig.2b	3.39	4.50	5.65	6.91	5.39
Normalized Ratio r_i	0.63	0.84	1.08	1.28	1.00
uniformed displacement applied to Fig.2c(mm)	0.10	0.10	0.10	0.10	0.10
stress concentration factor of Fig.2c	6.83	4.98	3.79	3.31	4.08
Normalized ratio r_i	1.67	1.22	0.93	0.81	1.00

Notes:
The uniformed displacement was applied to the partition wall, as seen in Fig.2a-2c. The calculation was carried out by ABAQUS, where the hexagonal eight-node element is used, as seen in Fig.S2. The average stress was obtained by $\sigma_{avg} = \frac{F}{S}$, where F is the reaction force. And the stress concentration factor k is obtained via $k = \frac{\sigma_{\max}}{\sigma_{avg}}$. Normalized ratio r_i is obtained via $r_{\mathrm{i}} = \frac{k_{\mathrm{i}}}{k_{7hole}}$.

Table S2: the elastic behavior under bending for different eccentricities.

porosity	35%				
eccentricity	0.87	0.77	0.6	0(5 hole)	0(7 hole)
semi-major axis(mm)	49.06	43.21	38.78	34.69	29.32
semi-minor axis(mm)	24.53	27.83	31.03	34.69	29.32
minimum width(mm)	109.45	167.91	212.18	253.13	189.57
minimum height(mm)	40.94	34.35	27.95	20.63	31.37
pressure applied of Fig.2d(KPa)	1.38	1.38	1.38	1.38	1.38
maximum bending stress(MPa)	0.62	0.63	0.65	0.69	0.64
normalized ratio r_i	0.97	0.98	1.02	1.07	1.00

Notes:
General technical requirements for the use of light rails for building partitions JG/T 169-2016[1] stipulates that the positive bending load capacity is measured by the uniform load method, i.e., the loading pressure is uniform. In this numerical experiment, the maximum stress of the cross section of the partition wall is analyzed when the partition wall is stacked. The magnitude of the pressure is given by Eqs. S20 and S21.
The uniformed pressure applied to the circular cavities partition wall is given by：

$$q_r = \rho \cdot V_r \cdot G / S \tag{S20}$$

The uniformed pressure applied to the elliptical cavities partition wall is given by：

$$q_e = \rho \cdot V_e \cdot G / S \tag{S21}$$

Where ρ is the density of the concrete partition wall, which is 2400kg/m^3; V_r, V_e is the volume; G is Gravity acceleration, which is $9.8 N/kg$; S is the surface area, which is $1.44 \times 10^6 \text{mm}^2$. The maximum bending stress (tension) occurs at the bottom of the wall.

Table S3: Hanging test, i.e., contact behavior between the screw and the wall for different eccentricities.

porosity		35%				
eccentricity		0.87	0.77	0.6	0(5 hole)	0(7 hole)
semi-major axis(mm)		49.06	43.21	38.78	34.69	29.32
semi-minor axis(mm)		24.53	27.83	31.03	34.69	29.32
minimum width(mm)		109.45	167.91	212.18	253.13	189.57
minimum height(mm)		40.94	34.35	27.95	20.63	31.37
point load applied of Fig.2e(N)		100.00	100.00	100.00	100.00	100.00
maximum contact stress (MPa)	top	3.57	6.61	10.17	13.74	7.89
	bottom	8.33	12.62	15.43	18.01	13.97
normalized ratio r_i		0.60	0.90	1.11	1.29	1.00

Notes:

The screw is simulated as a cylinder with the length of 50 mm (cantilever length is 25mm), diameter of 10 mm. The contact element used here is C3D8R. The point load is applied to the end of the cylinder. The maximum top and bottom stress from the contact area between the screw and partition wall was extracted. The normalized ratio is obtained via $r_i = \frac{\sigma_i}{\sigma_{7hole}}$.

Table S4: ultimate strength based on the loading Fig.2a-2c for different eccentricities.

porosity	35%				
eccentricity	0.87	0.77	0.6	0(5 hole)	0(7 hole)
semi-minor axis(mm)	49.06	43.21	38.78	34.69	29.32
semi-major axis(mm)	24.53	27.83	31.03	34.69	29.32
minimum width(mm)	109.45	167.91	212.18	253.13	189.57
minimum height(mm)	40.94	34.35	27.95	20.63	31.37
uniformed displacement applied to Fig.2a (mm)	4.00	4.00	4.00	4.00	4.00
ultimate strength of Fig.2a (MPa)	4.65	4.67	4.67	4.68	4.68
normalized Ratio r_i	0.99	1.00	1.00	1.00	1.00
uniformed displacement applied to Fig.2b(mm)	2.00	2.00	2.00	2.00	2.00
ultimate strength of Fig.2b (MPa)	3.84	3.25	2.74	1.99	3.08
normalized Ratio r_i	1.25	1.06	0.89	0.65	1.00
uniformed displacement applied to Fig.2c(mm)	0.18	0.18	0.18	0.18	0.18
ultimate strength of Fig.2c (MPa)	1.89	2.89	3.59	4.05	3.27
normalized ratio r_i	0.58	0.89	1.10	1.24	1.00

Note:

The uniformed displacement is applied to the partition wall until it reaches to 0.2% strain. The ultimate strength is expressed as $\sigma_{ult} = \frac{F_{\max}}{S}$, where F_{max} is the reaction force at the point of 0.2% strain, S is the surface area. And normalized ratio is calculated by $r = \frac{\sigma_{ult}}{\sigma_{7hole}}$

Table S5: the ultimate bending strength for different eccentricities.

porosity	35%				
eccentricity	0.87	0.77	0.6	0(5 hole)	0(7 hole)
semi-minor axis(mm)	49.06	43.21	38.78	34.69	29.32
semi-major axis(mm)	24.53	27.83	31.03	34.69	29.32
minimum width(mm)	109.45	167.91	212.18	253.13	189.57
minimum height(mm)	40.94	34.35	27.95	20.63	31.37
ultimate bending load(kN·m)	719.68	693.15	684.46	645.37	692.72
normalized ratio r_i	1.05	1.01	1.00	0.94	1.00

Note:

The uniformed pressure is gradually applied to the surface until the maximum stress reach the uniaxial strength 0.91 MPa (tension). And the normalized ratio is calculated by $r = \frac{\sigma_{ult}}{\sigma_{7hole}}$.

Table S6: the ultimate contact strength for different eccentricities.

porosity	35%				
eccentricity	0.87	0.77	0.6	0(5 hole)	0(7 hole)
semi-minor axis(mm)	49.06	43.21	38.78	34.69	29.32
semi-major axis(mm)	24.53	27.83	31.03	34.69	29.32
minimum width(mm)	109.45	167.91	212.18	253.13	189.57
minimum height(mm)	40.94	34.35	27.95	20.63	31.37
ultimate contact strength (N)	86.49	57.05	46.66	39.98	51.54
normalized Ratio r_i	1.68	1.11	0.91	0.78	1.00

Note:

Friction is not considered in this case. We gradually increased the point load until the maximum stress reached the uniaxial strength 7.2 MPa. And this force is considered as the ultimate contact load.

1.2 Porosity effect

Table S7: stress concentration factor under the loading based on Fig.2a-2c for different porosities.

porosity		25%	30%	35%	40%	50%
EP	eccentricity	0.77	0.77	0.77	0.77	0.77
	semi-major axis(mm)	36.52	40.00	43.21	46.19	51.64
	semi-minor axis(mm)	23.52	25.76	27.83	29.75	33.26
	minimum width(mm)	234.81	199.96	167.91	138.07	83.55
	minimum height(mm)	42.96	38.47	34.35	30.50	23.48
	uniformed displacement applied to Fig.2a (mm)	1.00	1.00	1.00	1.00	1.00
	stress concentration factor of top 2a	1.78	1.90	2.04	2.22	2.62
	uniformed displacement applied to Fig.2b (mm)	1.00	1.00	1.00	1.00	1.00
	stress concentration factor of Fig. 2b	3.49	3.79	4.42	4.86	6.76
	uniformed displacement applied to Fig.2c (mm)	0.10	0.10	0.10	0.10	0.10
	stress concentration factor of Fig.2c	3.61	4.41	4.98	6.02	9.41
CP	eccentricity	0(7 holes)	0(7 holes)	0(7 holes)	0(7 holes)	0(7 holes)
	semi-diameter(mm)	24.78	27.14	29.32	31.34	35.04
	minimum width(mm)	253.13	220.02	189.57	161.24	109.45
	minimum height(mm)	40.45	35.72	31.37	27.32	19.92
	uniformed displacement applied to Fig.2a (mm)	1.00	1.00	1.00	1.00	1.00
	stress concentration factor of Fig.2a	1.78	1.88	2.01	2.20	2.64
	uniformed displacement applied to Fig.2b (mm)	1.00	1.00	1.00	1.00	1.00
	stress concentration factor of Fig.2b	3.69	4.34	5.39	6.19	8.32
	uniformed displacement applied to Fig.2c (mm)	0.10	0.10	0.10	0.10	0.10
	stress concentration factor of Fig.2c	3.06	3.43	4.08	4.59	6.57

Table S8: the elastic behavior under bending for different porosities.

porosity		25%	30%	35%	40%	50%
EP	eccentricity	0.77	0.77	0.77	0.77	0.77
	semi-major axis(mm)	36.52	40.00	43.21	46.19	51.64
	semi-minor axis(mm)	23.52	25.76	27.83	29.75	33.26
	minimum width(mm)	234.81	199.96	167.91	138.07	83.55
	minimum height(mm)	42.96	38.47	34.35	30.50	23.48
	pressure applied to Fig.2d(KPa)	1.59	1.48	1.38	1.27	1.06
	Stress under bending(MPa)	0.66	0.65	0.63	0.60	0.55
CP	eccentricity	0(7 hole)	0(7 hole)	0(7 hole)	0(7 hole)	0(7 hole)
	semi-diameter(mm)	24.78	27.14	29.32	31.34	35.04
	minimum width(mm)	253.13	220.02	189.57	161.24	109.45
	minimum height(mm)	40.45	35.72	31.37	27.32	19.92
	pressure applied to Fig.2d(KPa)	1.59	1.48	1.38	1.27	1.06
	Stress under bending(MPa)	0.68	0.67	0.64	0.63	0.59

Table S9: Hanging test, i.e., contact behavior between the screw and the wall for different porosities.

porosity			25%	30%	35%	40%	50%
EP	eccentricity		0.77	0.77	0.77	0.77	0.77
	semi-major axis(mm)		36.52	40.00	43.21	46.19	51.64
	semi-minor axis(mm)		23.52	25.76	27.83	29.75	33.26
	minimum width(mm)		234.81	199.96	167.91	138.07	83.55
	minimum height(mm)		42.96	38.47	34.35	30.50	23.48
	point load applied to Fig.2e(N)		100.00	100.00	100.00	100.00	100.00
	contact stress (MPa)	top	3.69	4.91	6.61	8.67	15.25
		bottom	10.85	11.20	12.62	14.86	20.68
CP	eccentricity		0(7 hole)	0(7 hole)	0(7 hole)	0(7 hole)	0(7 hole)
	semi-diameter(mm)		24.78	27.14	29.32	31.34	35.04
	minimum width(mm)		253.13	220.02	189.57	161.24	109.45
	minimum height(mm)		40.45	35.72	31.37	27.32	19.92
	point load applied to Fig.2e(N)		100.00	100.00	100.00	100.00	100.00
	contact stress (MPa)	top	4.21	5.80	7.85	10.09	18.33
		bottom	11.73	12.31	13.65	16.11	22.96

Table S10: ultimate strength based on the loading Fig.2a-2c for different porosities.

porosity		25%	30%	35%	40%	50%
EP	semi-major axis(mm)	36.52	40.00	43.21	46.19	51.64
	semi-minor axis(mm)	23.52	25.76	27.83	29.75	33.26
	minimum width(mm)	234.81	199.96	167.91	138.07	83.55
	minimum height(mm)	42.96	38.47	34.35	30.50	23.48
	ultimate strength for the loading Fig.2a (MPa)	5.40	5.03	4.67	4.31	3.60
	ultimate strength for the loading Fig.2b (MPa)	4.09	3.64	3.25	2.89	2.07
	ultimate strength for the loading Fig.2c (MPa)	4.24	3.52	2.90	2.32	1.34
CP	semi-diameter(mm)	24.78	27.14	29.32	31.34	35.04
	minimum width(mm)	253.13	220.02	189.57	161.24	109.45
	minimum height(mm)	40.45	35.72	31.37	27.32	19.92
	ultimate strength for the loading Fig.2a (MPa)	5.42	5.05	4.68	4.33	3.59
	ultimate strength for the loading Fig.2b (MPa)	4.06	3.54	3.08	2.54	1.82
	ultimate strength for the loading Fig.2c (MPa)	4.52	3.92	3.27	2.68	1.55

Table S11: the ultimate bending strength for different porosities

porosity		25%	30%	35%	40%	50%
EP	semi-major axis(mm)	36.52	40.00	43.21	46.19	51.64
	semi-minor axis(mm)	23.52	25.76	27.83	29.75	33.26
	minimum width(mm)	234.81	199.96	167.91	138.07	83.55
	minimum height(mm)	42.96	38.47	34.35	30.50	23.48
	ultimate bending load (kN·m)	745.53	713.49	693.15	668.16	612.27
CP	semi-diameter(mm)	24.78	27.14	29.32	31.34	35.04
	minimum width(mm)	253.13	220.02	189.57	161.24	109.45
	minimum height(mm)	40.45	35.72	31.37	27.32	19.92
	ultimate bending load (kN·m)	744.38	707.79	683.78	649.29	584.21

Table S12: the ultimate contact strength for different porosities

porosity		25%	30%	35%	40%	50%
EP	semi-major axis(mm)	36.52	40.00	43.21	46.19	51.64
	semi-minor axis(mm)	23.52	25.76	27.83	29.75	33.26
	minimum width(mm)	234.81	199.96	167.91	138.07	83.55
	minimum height(mm)	42.96	38.47	34.35	30.50	23.48
	ultimate contact load(N)	66.36	64.29	57.05	48.45	34.82
CP	semi-diameter(mm)	24.78	27.14	29.32	31.34	35.04
	minimum width(mm)	253.13	220.02	189.57	161.24	109.45
	minimum height(mm)	40.45	35.72	31.37	27.32	19.92
	ultimate contact load(N)	61.38	58.49	52.75	44.69	31.36

1.3 Thermal performance

Table S13: Effect of the eccentricity

porosity	eccentricity	semi-major axis(mm)	semi-minor axis(mm)	minimum width(mm)	Hfl(J/s)
35%	0.87	49.06	24.53	109.45	79.78
	0.77	43.21	27.83	167.91	95.49
	0.6	38.78	31.03	212.18	111.04
	0(5 hole)	34.69	34.69	253.13	115.01
	0(7 hole)	29.32	29.32	189.57	106.25

Note:

The 2D Finite element method is performed by ABAQUS. Hfl represents the maximum heat flux through the surface.

Table S14: Porosity effect on the thermal performance.

porosity		25%	30%	35%	40%	50%
EP	eccentricity	0.77	0.77	0.77	0.77	0.77
	semi-major axis(mm)	36.52	40	43.21	46.19	51.64
	semi-minor axis(mm)	23.52	25.76	27.83	29.75	33.26
	minimum width(mm)	234.81	199.96	167.91	138.07	83.55
	Hfx(J/s)	116.47	108.09	95.5	83.75	59.05
CP	eccentricity	0(7 hole)	0(7 hole)	0(7 hole)	0(7 hole)	0(7 hole)
	semi-diameter(mm)	24.78	27.14	29.32	31.34	35.04
	minimum width(mm)	253.13	220.02	189.57	161.24	109.45
	Hfx(J/s)	124.89	119.37	106.25	96.94	75.63
Minimum width ratio(CP)/EP		1.08	1.1	1.13	1.17	1.31
Hfx ratio(CP)/EP		1.07	1.1	1.11	1.16	1.28
Improving(CP-EP)/EP		0.07	0.10	0.11	0.16	0.28

Note:

The 2D Finite element method is performed by ABAQUS.

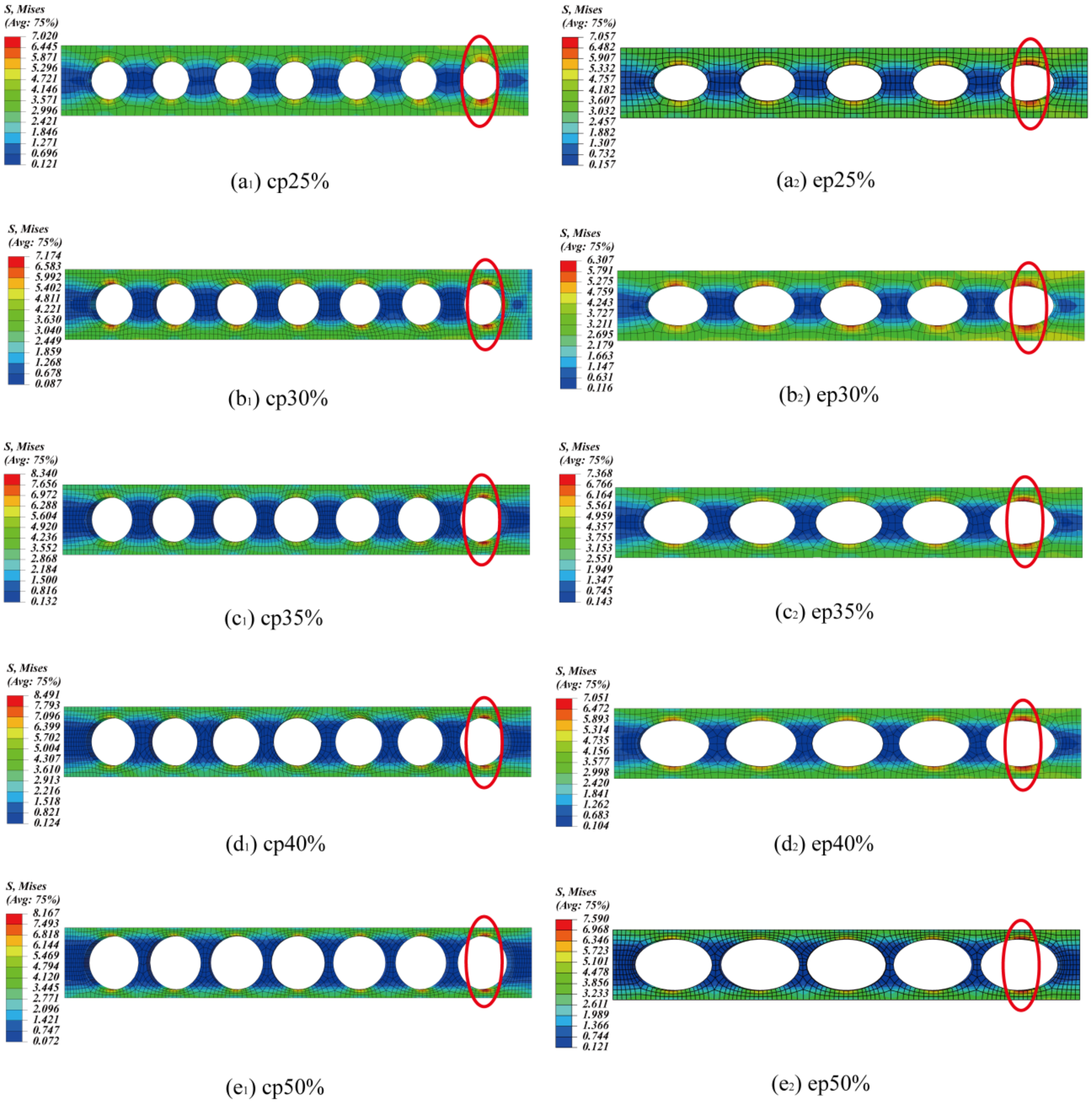

Fig.S1 Example of the stress concentration (indicated by the red circle) location for different porosities of loading for Fig.2b

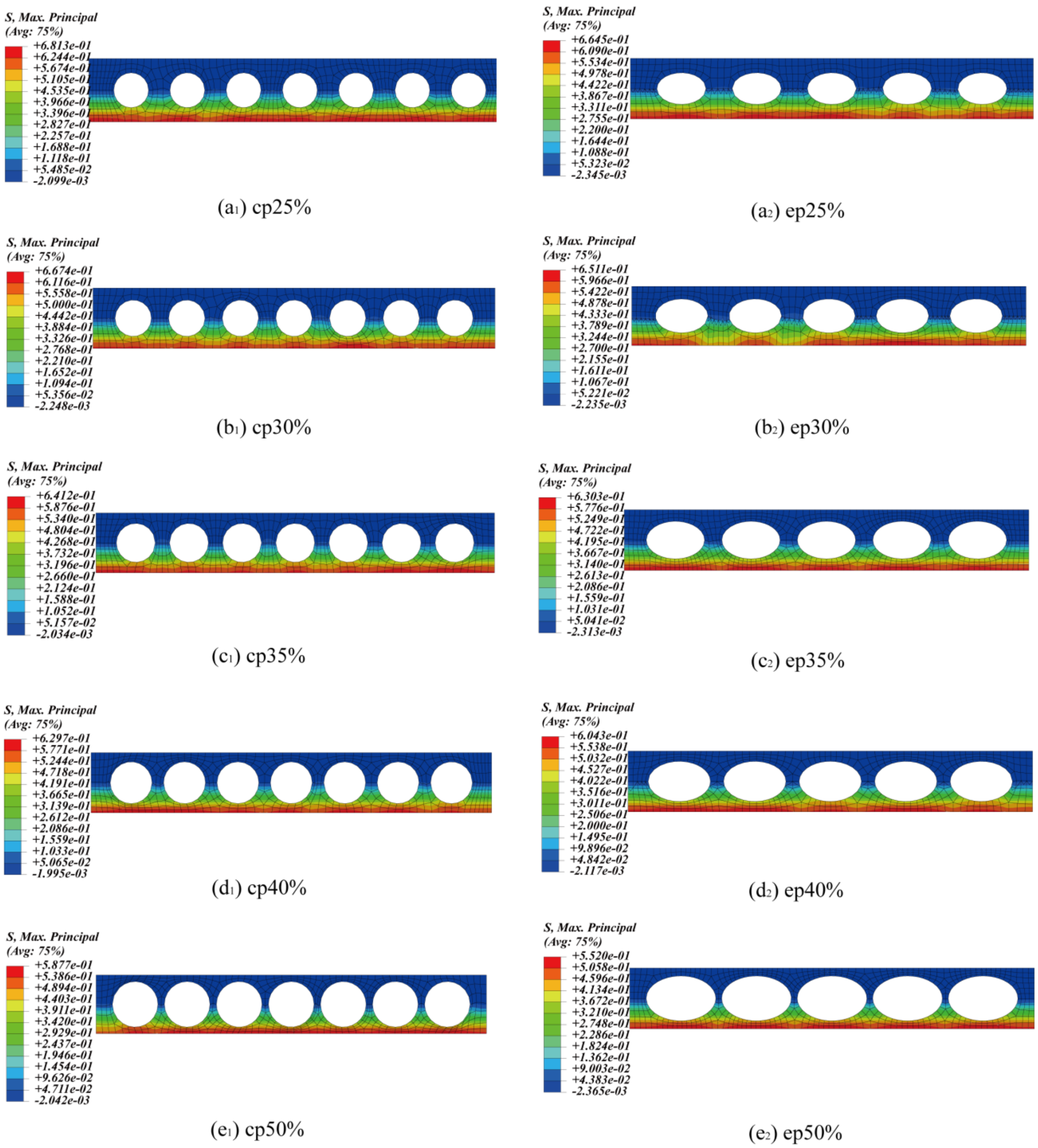

Fig.S2 bending stress location for different porosities.

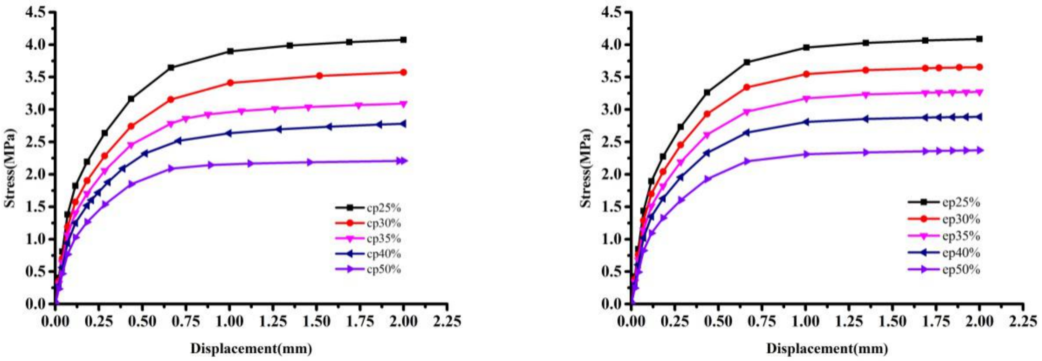

(a) Circular cavities panel (b) elliptical cavities panel

Fig.S3 Example of ealstic-plastic behaivior of loading for Fig.2b.
